# Simulation-based power and sample size calculation for designing interrupted time series analyses of count outcomes in evaluation of health policy interventions

**DOI:** 10.1016/j.conctc.2019.100474

**Published:** 2019-10-16

**Authors:** Wei Liu, Shangyuan Ye, Bruce A. Barton, Melissa A. Fischer, Colleen Lawrence, Elizabeth J. Rahn, Maria I. Danila, Kenneth G. Saag, Paul A. Harris, Stephenie C. Lemon, Jeroan J. Allison, Bo Zhang

**Affiliations:** a School of Management, Harbin Institute of Technology, Harbin, Heilongjiang 150001, China; b Department of Population Medicine, Harvard Pilgrim Health Care Institute and Harvard Medical School, Boston, MA, 02115, USA; c Department of Internal Medicine, University of Massachusetts Medical School, Worcester, MA, 01605, USA; d Meyers Primary Care Institute, University of Massachusetts Medical School, Fallon Foundation, and Fallon Community Health Plan, Worcester, MA, 01605, USA; e Vanderbilt Institute for Clinical and Translational Research, Vanderbilt University Medical Center, Nashville, TN, 37232, USA; f Division of Clinical Immunology and Rheumatology, University of Alabama at Birmingham, Birmingham, AL, 35294, USA; g Department of Biomedical Informatics and Department of Biomedical Engineering, Vanderbilt University, Nashville, TN, 37203, USA; h Department of Neurology and ICCTR Biostatistics and Research Design Center, Boston Children’s Hospital and Harvard Medical School, Boston, MA, 02215, USA

**Keywords:** Policy evaluation, Interrupted time series, Count outcomes, Segmented regression, Quasi-experimental design, Power, Sample size calculation

## Abstract

**Objective:**

The purpose of this study was to present the design, model, and data analysis of an interrupted time series (ITS) model applied to evaluate the impact of health policy, systems, or environmental interventions using count outcomes. Simulation methods were used to conduct power and sample size calculations for these studies.

**Methods:**

We proposed the models and analyses of ITS designs for count outcomes using the Strengthening Translational Research in Diverse Enrollment (STRIDE) study as an example. The models we used were observation-driven models, which bundle a lagged term on the conditional mean of the outcome for a time series of count outcomes.

**Results:**

A simulation-based approach with ready-to-use computer programs was developed to calculate the sample size and power of two types of ITS models, Poisson and negative binomial, for count outcomes. Simulations were conducted to estimate the power of segmented autoregressive (AR) error models when autocorrelation ranged from −0.9 to 0.9, with various effect sizes. The power to detect the same magnitude of parameters varied largely, depending on the testing level change, the trend change, or both. The relationships between power and sample size and the values of the parameters were different between the two models.

**Conclusion:**

This article provides a convenient tool to allow investigators to generate sample sizes that will ensure sufficient statistical power when the ITS study design of count outcomes is implemented.

## 1 Introduction

Interrupted time series (ITS) analysis is a strong quasi-experimental design that can be used to evaluate the effectiveness of a population-level intervention that is clearly defined at a given time point ([[1], [2], [3]]). ITS designs usually involve repeatedly collecting a particular aggregate level outcome pre- and post-intervention ([4,5]). The segmented time series regression model ([2]) with one discontinuity time point is the general tool used to evaluate such data, in which each segment can have a different level, trend, or both. That is, two-line segments are fitted simultaneously and separated at the intervention time point. A change in the “level” of the outcome is indicated by a discontinuity at the time point when the intervention was introduced, and the change in the “trend” is revealed by a change of slope. Statistical hypothesis tests [6] are typically used to detect changes in outcome after the implementation of intervention. ITS is typically used when randomized trials are infeasible and has been extensively used on evaluating public health and health service interventions ([3,7]). The assumptions and advantages of using ITS analysis have been thoroughly discussed ([8,9]). Although most studies have focused on aggregated level single-arm ITS design, two-arm ITS design ([4]) and individual level ITS models ([10]) have also been discussed.

Modeling the time series of the observed count data is a more challenging task than creating time series models for continuous data. Unlike modeling a normal time series of continuous data, according to Jung et al. [11], a potential model for the time series of the count data must be able to characterize both the dependence structure and the overdispersion of data. Several models have been proposed and categorized into two types ([12]): observation-driven models, which bundle a lagged term on the conditional mean of the outcome; and parameter-driven models, driven by a dynamic process, which are reviewed by Cameron and Trivedi [13]. That is, observation-driven models directly model the conditional mean of current count data to historical data, and parameter-driven models can be considered to be a generalized linear model (GLM) with a pre-specified dependence structure.

For the observation-driven models, the two most commonly used models are the generalized linear autoregressive moving average (GLARMA) model and the log-linear (LL) model. The GLARMA model was proposed by Shephard [14] and Davis et al. [15], and the LL model, first proposed by Zeger and Qaqish [16], has been further investigated by Fokianos and Fried [17,18], Woodard, Mateeson, and Henderson [19] and Douc, Doukhan, and Moulines [20]. Further discussion on theoretical properties, like the stationarity and ergodicity of the GLARMA and LL models, can be found in Dunsmuir and Scott [21] and Liboschik et al. [22]. The most common parameter-driven model is the Zeger model [23]. Considering the Gaussian linear process of the conditional mean of the outcome, the Zeger model was studied by Zeger [23] and Davis et al. [24]. Its equivalent logarithmic form was studied by Chan and Ledorter [25], Kuk and Cheng [26], Jung and Liesenfeld [27], and Jung and Tremayne [28].

Though count outcomes are the common practice in policy research, the ITS design of count outcomes has only made limited appearances in the literature. For instance, Walter et al. [29] modeled injury count data using the negative binomial log-linear model and fit the model by the maximum likelihood estimator. Wang, Olivier, and Grzebieta [30] considered the same model and compared the estimation performance between the maximum likelihood estimator, the full Bayesian estimator, and the empirical Bayesian estimator via simulation. However, the power of the statistical tests in ITS analyses with count data has never been studied. To address this gap, in this manuscript we conducted simulations to estimate the power and sample sizes in various settings. Here, we only considered the most basic two-phase single-arm ITS design for count outcomes. More complicated three-phase two-arm models are beyond the scope of this paper. A similar study on the two-phase ITS design of continuous data outcomes was conducted by Zhang, Wagner, and Ross-Degnan [6]. Herein, we solely focus on the observation-driven model for a time series of count data, in particular the LL models. We only consider the observation-driven models because they are designed to allow the likelihood to be evaluated easily, but the parameter-driven models usually involves high-dimensional integration, which is computationally infeasible [15].

## 2 Exemplar study: Strengthening Translational Research in a Diverse Enrollment (STRIDE) study

The power and sample size calculation for the ITS design of count outcomes were motivated by the required statistical analysis of data generated from the STRIDE study, an ongoing five-year study aimed at developing an intervention to increase the engagement of African Americans and Latinos in translational research ([31]). Since the primary outcome of the study is the number of African Americans and Latinos enrolled in ongoing translational clinical trials, to mitigate their historical underrepresentation in translational research, the STRIDE study is a representative example of the ITS design of count data.

The STRIDE project is a partnership of the CTSAs (Clinical and Translational Science Awards program) at the University of Massachusetts Medical School, the University of Alabama at Birmingham, and Vanderbilt University—three geographically diverse sites with large African American and Latino populations. The STRIDE intervention was motivated by previous studies of exposed barriers to research participation ([[32], [33], [34]]). Participant and systematic barriers include limited research literacy, lack of trust stemming from historical abuses, lack of research staff training in appropriate cultural competency skills, and confusion of informed consent procedures in research. To overcome these barriers, the proposed multi-level intervention contains three components: (1) storytelling for the promotion of research literacy; (2) simulation-based training to improve culturally appropriate recruitment and informed consent; and (3) an electronic consent platform to enhance cultural competency. The STRIDE intervention builds synergistically on emerging work at each institution to create a new intervention that addresses barriers on multiple levels. The primary outcome of the STRIDE project is the number of recruitments of African Americans and Latinos, as well as the total recruitment.

To test the effectiveness of the STRIDE intervention, we have recruited ongoing translational clinical studies at each of the three partnering CTSA hubs. Both the interventions and contemporaneous controls (i.e., clinical trials without STRIDE intervention) are introduced at each of the CTSA hubs. Each participating university layers the STRIDE intervention on one study, with another study serving as the un-intervened control. Thus, using the number of African American and Latino participants recruited, or the total number of participants, as the prime response variable (outcome), the STRIDE intervention will be evaluated by the two-arm ITS design and will include six ongoing translational research studies. Three studies will receive the intervention and comprise the study group, and the remaining three un-intervened studies will comprise the comparison group. The study outcomes are collected on a weekly basis. The change in study outcomes will be examined based on a two-phase framework (pre-implementation versus post implementation).

## 3 Methods

### 3.1 Design and analysis of a single-arm ITS study with count outcomes

The STRIDE study has motivated our investigation of a time series study design. In a two-phase ITS study, if all study subjects and sites are planned to be exposed to an intervention over time, then such a study is a single-arm ITS study. Let $Y_{t}$ represent the count outcome variable that is measured at time point t, let $T_{t}$ be the actual or converted study time (in the simulation, we also considered the logarithm of the actual time to avoid model explosion) from the start to the end of the study, let $X_{t}$ be a binary indicator for the second phase of the study, and let $t_{0}$ be the time point after the onset of intervention.

### 3.2 Observation-driven model

Here, we give a brief introduction of the modeling framework for the observation-driven segment regression time series model of count outcomes. For a single-arm ITS design of count outcomes, a common kind of observation-driven time series build model on the logarithm of the conditional mean of the response $Y_{t}$ can be written as

(1) $$\ln\left(\mu_{t}\right)=\beta_{0}+\beta_{1}T_{t}+\beta_{2}X_{t}+\beta_{3}\left(T_{t}-t_{0}\right)X_{t}+g\left(F_{t-1};\theta \right)$$

where $F_{t-1}=\left\{Y_{0},\cdots ,Y_{t-1\;},\;\;\mu_{0},\cdots ,\mu_{t-1}\right\}$, $\mu_{t}=E\left(Y_{t}|F_{t-1}\right)$ is the mean of $Y_{t}$ conditioning on the past responses and means, the function g joints current outcome with past outcomes that are correlated in the time series, $T_{t}$ is the actual time of the study, $t_{0}$ is the time point of intervention, $X_{t}$ is the binary indicator for the second phase of the study, and $\beta_{0},\beta_{1},\beta_{2},\beta_{3}$, and θ are unknown parameters.

In observation-driven models, the effect of covariates on the outcome or its mean is complicated and difficult to interpret because the conditional mean also dependents on past outcomes ([15]). For the ITS design, the coefficient $\beta_{0}$ is the regression intercept representing the starting level of the logarithm of the conditional mean, $\beta_{1}$ is the slope of the logarithm of the conditional mean before the implementation of the intervention, $\beta_{2}$ represents the change in the level of the logarithm of the conditional mean caused by the intervention versus non-intervention, and $\beta_{3}$ represents the difference in the slopes of the logarithm of the conditional mean caused by the intervention versus non-intervention. The focus of the ITS analysis is to examine the significance of $\beta_{2}$, which indicates an immediate intervention effect on the level change of the conditional mean, and the significance of$\;\beta_{3}$, which indicates the intervention effect in terms of the change in the trend of the conditional mean. Note that the purpose of subtracting $t_{0}$, the time point after the onset of intervention, from the study time $T_{t}$ is to maintain the interpretation of the corresponding regression coefficients $\beta_{3}$.

Let $\eta_{t}=\beta_{0}+\beta_{1}T_{t}+\beta_{2}X_{t}+\beta_{3}\left(T_{t}-t_{1}\right)X_{t}$, while p and q are non-negative integers less than t. A variety of choices for g were proposed. For example, when $g\left(F_{t-1}\right)=\theta \;\ln\;Y_{t-1}^{*}-\eta_{t-1}$, (model (1)) is the Zeger–Qaqish model [16], where $Y_{t-1}^{*}$ is a transition of the $Y_{t-1}$ shielding influence from a zero value, such as $Y_{t-1}^{*}=\max\left\{Y_{t-1},c\right\}$, with s positive constant c; when $g\left(F_{t-1}\right)=Z_{t}$ , where $Z_{t}=\overset{p}{\underset{j=1}{\sum }}\alpha_{j}\left(Z_{t-j}+e_{t-j}\right)+\overset{q}{\underset{j=1}{\sum }}\gamma_{j}e_{t-j}$ where $e_{t}=\frac{Y_{t}-\mu_{t}}{\nu_{t}}$ is a scaling residual and $\nu_{t}$ is some scaling function of $\mu_{t}$, and θ={all $\alpha_{j}$ and $\gamma_{j}\}$, the model is a generalized linear autoregressive moving average (GLARMA) model [14]; when $g\left(F_{t-1}\right)=\overset{p}{\underset{j=1}{\sum }}\alpha_{j}\;\ln\left(\mu_{t-j}\right)\;+\overset{q}{\underset{j=1}{\sum }}\gamma_{j}\;\ln\left(Y_{t-j}+1\right)$, it is a log-linear (LL) model. Here, we will focus on LL models with low orders, i.e., small values of p and q. Specifically, we model the time series of counts via the LL model with p=0 and q=1, denoted by LL (0,1), which has the form

(2) $$\ln\left(\mu_{t}\right)=\eta_{t}+\;\gamma_{1}\;\ln\left(Y_{t-1}+1\right).$$

Where the logarithm of the mean linearly depends on the logarithm of the last observation, which positively or negatively depends on $\gamma_{1}$. Since we use some logarithm functions in this model, it is hard to develop formulas for the mean or the autocovariance function of $\ln\mu_{t}$ or $Y_{t}$.

The most commonly used distribution for count data is Poisson distribution, in which the conditional distribution of response $Y_{t}$ on past history $F_{t-1}$ is denoted by $Y_{t}|F_{t-1}\sim Poisson\left(\mu_{t}\right)$, and the density has the form

(3) $$P\left(Y_{t}=y\text{|}F_{t-1}\right)=\frac{\exp\left(-\mu_{t}\right)}{\text{Γ}\left(y+1\right)}\mu_{t}^{y}$$

Poisson distribution is simple and popular. However, Poisson distribution is known to have equal mean and variance, which can be unrealistic in some settings. A more appropriate and flexible model for modeling count data with a larger overdispersion than Poisson (i.e., with greater variability) is negative binomial distribution. Denoting the conditional distribution of response $Y_{t}$ on past history $F_{t-1}$ to be $Y_{t}|F_{t-1}\sim NB\left(\mu_{t},\varphi \right)$, the density function for negative binomial can be expressed as

(4) $$P\left(Y_{t}=y\text{|}F_{t-1}\right)=\frac{\text{Γ}\left(\varphi +y\right)}{\text{Γ}\left(y+1\right)\text{Γ}\left(\varphi \right)}{\left(\frac{\varphi }{\varphi +\mu_{t}}\right)}^{\varphi }{\left(\frac{\mu_{t}}{\varphi +\mu_{t}}\right)}^{y}$$

where φ>0, with variance $\mu_{t}+\frac{\mu_{t}^{2}}{\varphi }$.

For many observation-driven models of count time series, the stationarity and ergodicity of the process, which are used to develop consistency and asymptotic normality, are only partially discussed in some special and simple scenarios, the majority of which are still unclear. For Poisson responses with $\eta_{t}=\eta_{0}$ (constant), model (2) has a stationary distribution when $\left|\gamma_{1}\right|<1$ . More discussion on the stationarity and ergodicity of GLARMA and LL models can be found in Dunsmuir and Scott [21] and Liboschik et al. [22].

### 3.3 Simulation-based sample size and power calculation

We used a simulation-based method to calculate the power of different statistical tests under different scenarios (different sample size and parameter values) for the two-phase single-arm ITS design of the count outcomes. For an arbitrary two-sided statistical test with the null hypothesis $H_{0}:\;\beta =0$ versus the alternative $H_{1}:\beta \neq 0$, where β can be either a univariate regression coefficient or any combination of multiple coefficients defined in Section 3.2. Here, we considered three null hypotheses in our simulation study: (i) $\beta_{2}=\beta_{3}=0$, to test whether any changes (level, trend or both) exist after intervention; (ii) $\beta_{2}=0$, to test the change on level after intervention; and (iii) $\beta_{3}=0$, to test any trend changes after intervention. In this simulation-based sample size and power calculation, we considered the logarithm of actual time to avoid model explosion. $\beta_{2}$ represented the change in the level of the logarithm of the conditional mean caused by intervention versus non-intervention, and $\beta_{3}$ represented the difference in the slopes of the logarithm of the conditional mean caused by intervention versus non-intervention. For these three hypothesis tests, chi-square (Wald) tests were employed as test statistics, and the empirical power of these tests were calculated via simulation.

For any statistical tests, the power under a pre-specified significance level is defined as the probability that rejecting the null hypothesis conditioning with the alternative hypothesis is true, i.e., $P\left(Reject\;H_{0}|H_{1}\;is\;true\right)$. Since this probability is generally unknown, we used simulation to estimate the power. For the simulation-based method, a large number of datasets were randomly generated from the ITS model we introduced in Section 3.2, with pre-specified non-zero coefficients, and statistical hypothesis tests were conducted for each dataset. Then, the empirical power was estimated as the frequency that the null hypothesis was rejected divided by the total number of datasets. Denoting R as the number of datasets, this estimated power will approach the true power if the R is large enough. In our simulation study, we used R=200 and a significance level of 0.05 for all cases.

We considered different scenarios for sample sizes, parameters, and correlation coefficients. For sample size n, i.e., the number of observations over time, we considered the cases n=18,24,32,48,56,64,80, and 96, with equal numbers of observations uniformly distributed before and after policy intervention. For the negative binomial distributions, we specified the overdispersion parameter to be φ=2. The start value $Y_{0}$ was set to be 0. We considered 3 hypothesis tests. For hypothesis test (i), we considered the different values of $\beta_{2}+\beta_{3}$, which are the expected level change plus the expected trend change after the intervention of conditioning on the same outcome history. In this case, we chose the parameter values to be ±0.25,±0.5 and ±1 for both the Poisson and negative binomial time series. For hypothesis test (ii), we considered the different values of $\beta_{2}$, which is the expected level change caused by the intervention of conditioning on the same outcome history. For this test, with $\beta_{3}$ specified to be 0, we chose the values of $\beta_{2}$ to be ±0.25,±0.5, and ±1 for the Poisson time series, and ±1,±2, and ±3 for the negative binomial time series. For hypothesis test (iii), we considered the different values of $\beta_{3}$, which is the expected trend change caused by the intervention of conditioning on the same outcome history. For this test, with $\beta_{2}$ specified to be 0, we chose the values of $\beta_{3}$ to be ±0.01,±0.05, and ±0.1 for the Poisson time series, and ±0.05,±0.1, and ±0.25 for the negative binomial time series. Negative values for the parameters indicate a “decrease” (either level, trend, or both) after intervention, and positive values indicate an “increase” after intervention. We chose different parameter values between the Poisson and negative binomial models because negative binomial models usually use modeling count data with larger overdispersion than Poisson models. We also considered different values for coefficient $\gamma_{1}$ in model (2), which represents the degree of dependence between the current conditional mean $\mu_{t}$ and historical outcomes. Here, we considered all cases from −0.9 to 0.9, with a step of 0.2 and case $\gamma_{1}=0$, which represents the case with no correlation.

## 4 Results

**Fig. 1:**
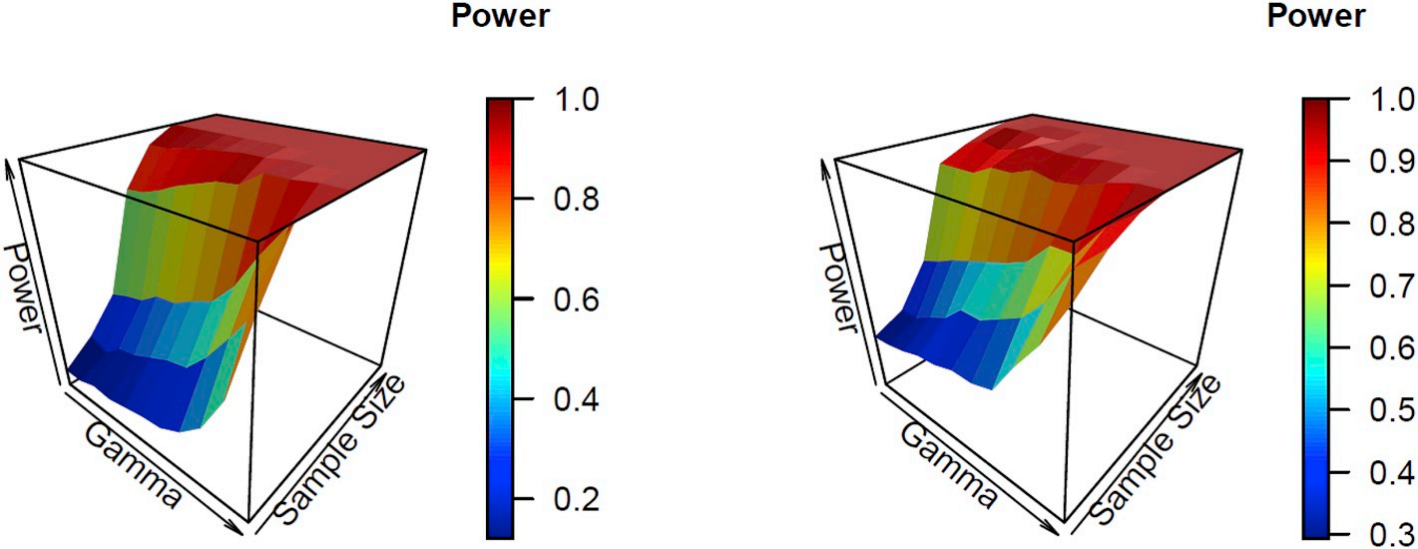
Surface plots of the estimated power for hypothesis test $\beta_{2}=\beta_{3}=0$ of $\gamma_{1}$ and sample size n. The left panel is for the Poisson time series with $\beta_{2}+\beta_{3}=-1$; the right panel is for the negative binomial time series with $\beta_{2}+\beta_{3}=-1$.

Table 1, Table 2 show the estimated power for testing hypothesis (i) $H_{0}:\beta_{2}=\beta_{3}=0$ for the Poisson and negative binomial time series for model (2), with $\beta_{2}+\beta_{3}=\pm 0.25,\;\pm 0.5,\;\pm 1,$ based on a significance level of 0.05. The estimated power increased as $\gamma_{1}$, the sample size increased, or the values of the parameter became more significant (i.e., the absolute value of $\beta_{2}+\beta_{3}$ became greater). The trends of the estimated power of $\gamma_{1}$ and sample size n are illustrated by the surface plots in Fig. 1.

**Table 1:** Estimated power testing $H_{0}:\beta_{2}=\beta_{3}=0$ for the Poisson time series with a conditional mean model LL (0,1) when $\beta_{2}+\beta_{3}$ = ±0.25, ±0.5, ±1 based on 200 simulated data sets and a statistical significance level of 0.05. The symbol “-” indicates that more than one fourth of the data sets cannot be successfully generated.

$\gamma_{1}$	Sample size							
	18	24	32	48	56	64	80	96
$\beta_{2}+\beta_{3}$ = −1								
−0.9	0.08	0.18	0.33	0.78	0.94	1	1	1
−0.7	0.08	0.14	0.36	0.79	0.94	1	1	1
−0.5	0.11	0.21	0.38	0.82	0.96	1	1	1
−0.3	0.10	0.23	0.44	0.86	0.97	1	1	1
−0.1	0.12	0.27	0.45	0.89	0.98	1	1	1
0	0.14	0.31	0.50	0.92	0.99	1	1	1
0.1	0.15	0.33	0.54	0.93	0.99	1	1	1
0.3	0.20	0.43	0.64	0.99	1	1	1	1
0.5	0.29	0.56	0.79	0.99	1	1	1	1
0.7	0.47	0.70	0.92	1	1	1	1	1
0.9	0.88	1	1	1	1	1	1	1
$\beta_{2}+\beta_{3}$ = −0.5								
−0.9	0.05	0.10	0.12	0.28	0.47	0.63	0.94	1
−0.7	0.04	0.10	0.13	0.32	0.49	0.66	0.95	1
−0.5	0.05	0.12	0.15	0.33	0.52	0.69	0.98	1
−0.3	0.07	0.13	0.20	0.38	0.60	0.75	0.99	1
−0.1	0.08	0.15	0.18	0.48	0.66	0.85	1	1
0	0.11	0.13	0.22	0.50	0.69	0.90	1	1
0.1	0.11	0.15	0.25	0.54	0.76	0.92	1	1
0.3	0.13	0.20	0.31	0.66	0.90	0.98	1	1
0.5	0.17	0.27	0.38	0.91	0.97	1	1	1
0.7	0.27	0.45	0.75	1	1	1	1	1
0.9	0.74	0.97	1	1	1	1	1	1
$\beta_{2}+\beta_{3}$ = −0.25								
−0.9	0.03	0.07	0.06	0.15	0.15	0.22	0.43	0.71
−0.7	0.04	0.06	0.05	0.15	0.19	0.23	0.54	0.79
−0.5	0.04	0.10	0.07	0.16	0.17	0.26	0.47	0.83
−0.3	0.07	0.10	0.09	0.19	0.17	0.27	0.60	0.86
−0.1	0.05	0.10	0.08	0.17	0.26	0.32	0.65	0.97
0	0.06	0.10	0.09	0.19	0.29	0.36	0.77	0.98
0.1	0.07	0.09	0.14	0.21	0.30	0.36	0.80	1
0.3	0.10	0.13	0.16	0.26	0.38	0.63	0.95	1
0.5	0.13	0.14	0.18	0.39	0.67	0.85	1	1
0.7	0.17	0.25	0.40	0.86	0.99	1	1	1
0.9	0.40	0.69	0.99	1	1	1	–	–
$\beta_{2}+\beta_{3}$ = 0.25								
−0.9	0.06	0.06	0.06	0.15	0.17	0.21	0.46	0.74
−0.7	0.04	0.08	0.10	0.13	0.17	0.24	0.45	0.76
−0.5	0.05	0.08	0.07	0.15	0.19	0.26	0.51	0.86
−0.3	0.05	0.10	0.06	0.18	0.18	0.32	0.63	0.89
−0.1	0.07	0.12	0.07	0.17	0.23	0.35	0.69	0.93
0	0.07	0.11	0.09	0.17	0.31	0.35	0.69	0.95
0.1	0.07	0.11	0.11	0.22	0.30	0.40	0.79	0.99
0.3	0.05	0.08	0.14	0.27	0.41	0.55	0.87	1
0.5	0.10	0.13	0.15	0.39	0.63	0.80	1	1
0.7	0.15	0.21	0.36	0.89	0.99	1	1	1
0.9	0.26	0.42	0.97	–	–	–	–	–
$\beta_{2}+\beta_{3}$ = 0.5								
−0.9	0.05	0.07	0.11	0.33	0.52	0.71	0.96	1
−0.7	0.04	0.10	0.13	0.35	0.57	0.71	0.93	1
−0.5	0.04	0.11	0.12	0.41	0.55	0.75	0.97	1
−0.3	0.07	0.12	0.17	0.47	0.64	0.86	0.99	1
−0.1	0.09	0.14	0.21	0.46	0.70	0.89	0.99	1
0	0.09	0.12	0.24	0.52	0.76	0.94	1	1
0.1	0.08	0.17	0.29	0.58	0.74	0.95	1	1
0.3	0.13	0.15	0.30	0.69	0.89	0.98	1	1
0.5	0.17	0.27	0.50	0.91	0.98	1	1	1
0.7	0.33	0.55	0.93	1	1	1	1	–
0.9	0.52	0.94	1	–	–	–	–	–
$\beta_{2}+\beta_{3}$ = 1								
−0.9	0.15	0.22	0.44	0.91	0.98	1	1	1
−0.7	0.16	0.24	0.48	0.93	0.99	1	1	1
−0.5	0.20	0.29	0.52	0.92	0.99	1	1	1
−0.3	0.21	0.36	0.56	0.96	0.99	1	1	1
−0.1	0.25	0.37	0.66	0.97	1	1	1	1
0	0.32	0.48	0.70	0.99	1	1	1	1
0.1	0.34	0.53	0.77	0.98	1	1	1	1
0.3	0.45	0.69	0.90	1	1	1	1	1
0.5	0.72	0.91	0.99	1	1	0.99	1	1
0.7	0.98	1	1	1	1	–	–	–
0.9	0.97	0.99	–	–	–	–	–	–

**Table 2:** Estimated power testing $H_{0}:\beta_{2}=\beta_{3}=0$ for the negative binomial time series with a conditional mean model LL (0,1) when $\beta_{2}+\beta_{3}$ = ±0.25, ±0.5, ±1 based on 200 simulated data sets and a statistical significance level of 0.05. The symbol “-” indicates that more than one fourth of the data sets cannot be successfully generated.

$\gamma_{1}$	Sample size							
	18	24	32	48	56	64	80	96
$\beta_{2}+\beta_{3}$ = −1								
−0.9	0.26	0.32	0.50	0.89	0.94	0.98	1	1
−0.7	0.27	0.32	0.53	0.89	0.97	1	1	1
−0.5	0.29	0.37	0.57	0.89	0.95	1	1	1
−0.3	0.32	0.41	0.60	0.93	0.97	0.99	1	1
−0.1	0.33	0.49	0.63	0.93	1.00	0.99	1	1
0	0.36	0.49	0.66	0.96	0.99	1	1	1
0.1	0.36	0.54	0.70	0.94	0.99	1	1	1
0.3	0.39	0.59	0.79	0.95	1	1	1	1
0.5	0.54	0.69	0.79	0.98	1	1	1	1
0.7	0.67	0.84	0.91	1	1	1	1	1
0.9	0.84	0.93	0.98	1	1	1	1	1
$\beta_{2}+\beta_{3}$ = −0.5								
−0.9	0.26	0.29	0.40	0.62	0.77	0.87	0.97	1
−0.7	0.28	0.31	0.43	0.65	0.79	0.87	0.97	1
−0.5	0.31	0.33	0.46	0.68	0.80	0.88	0.98	1
−0.3	0.33	0.36	0.50	0.72	0.86	0.90	0.97	1
−0.1	0.37	0.40	0.54	0.77	0.86	0.94	0.99	1
0	0.39	0.43	0.54	0.81	0.84	0.93	1	1
0.1	0.43	0.47	0.57	0.82	0.93	0.96	1	1
0.3	0.38	0.55	0.65	0.85	0.94	0.98	1	1
0.5	0.58	0.67	0.79	0.94	1.00	1	1	1
0.7	0.66	0.87	0.88	0.97	0.98	1	1	1
0.9	0.87	0.94	0.99	1	1	1	1	1
$\beta_{2}+\beta_{3}$ = −0.25								
−0.9	0.28	0.35	0.40	0.47	0.59	0.66	0.84	0.92
−0.7	0.29	0.37	0.40	0.52	0.55	0.60	0.89	0.95
−0.5	0.33	0.35	0.41	0.51	0.60	0.66	0.87	0.95
−0.3	0.36	0.38	0.47	0.57	0.73	0.77	0.88	0.95
−0.1	0.39	0.41	0.48	0.61	0.66	0.76	0.97	0.98
0	0.39	0.46	0.49	0.70	0.79	0.82	0.90	0.99
0.1	0.43	0.49	0.60	0.70	0.84	0.87	0.95	0.99
0.3	0.46	0.56	0.63	0.80	0.86	0.93	0.97	1
0.5	0.60	0.69	0.82	0.94	0.96	0.99	1	1
0.7	0.76	0.85	0.93	0.98	1.00	0.99	1	1
0.9	0.94	0.99	1	1	1	1	1	1
$\beta_{2}+\beta_{3}$ = 0.25								
−0.9	0.33	0.42	0.51	0.63	0.70	0.77	0.84	0.93
−0.7	0.33	0.43	0.49	0.66	0.71	0.81	0.89	0.95
−0.5	0.37	0.47	0.53	0.59	0.72	0.78	0.91	0.95
−0.3	0.42	0.47	0.55	0.67	0.79	0.86	0.94	0.96
−0.1	0.47	0.48	0.60	0.81	0.79	0.89	0.95	1
0	0.51	0.56	0.61	0.82	0.80	0.94	0.97	0.99
0.1	0.59	0.61	0.65	0.85	0.88	0.94	0.99	1
0.3	0.58	0.74	0.85	0.94	0.95	0.99	1	1
0.5	0.80	0.86	0.96	0.98	1	1	1	1
0.7	0.90	0.95	0.98	0.99	1	1	1	1
0.9	0.99	1	1	1	1	1	–	1
$\beta_{2}+\beta_{3}$ = 0.5								
−0.9	0.43	0.46	0.60	0.75	0.88	0.94	0.99	1
−0.7	0.44	0.52	0.56	0.75	0.88	0.94	0.99	1
−0.5	0.46	0.55	0.61	0.84	0.87	0.95	0.99	1
−0.3	0.49	0.57	0.66	0.83	0.92	0.97	0.98	1
−0.1	0.48	0.65	0.76	0.91	0.97	0.99	1	1
0	0.57	0.69	0.76	0.90	0.97	1	1	1
0.1	0.66	0.71	0.80	0.96	0.99	0.99	1	1
0.3	0.78	0.86	0.90	0.99	1	1	1	1
0.5	0.83	0.92	0.97	0.99	1	1	1	1
0.7	0.96	0.99	1	1	1	1	1	–
0.9	0.99	1	1	–	–	–	–	–
$\beta_{2}+\beta_{3}$ = 1								
−0.9	0.58	0.69	0.82	0.96	1	0.99	1	1
−0.7	0.62	0.67	0.85	0.98	1	1	1	1
−0.5	0.64	0.73	0.90	1.00	1	1	1	1
−0.3	0.62	0.80	0.86	0.99	1	1	1	1
−0.1	0.74	0.84	0.94	1	1	1	1	1
0	0.75	0.84	0.95	0.99	1	1	1	1
0.1	0.82	0.91	0.98	1	1	1	1	1
0.3	0.89	0.95	0.99	1	1	1	1	1
0.5	0.94	1	1	1	1	1	1	1
0.7	0.99	1	1	1	1	1	1	–
0.9	1	1	1	1	1	1	–	–

**Fig. 2:**
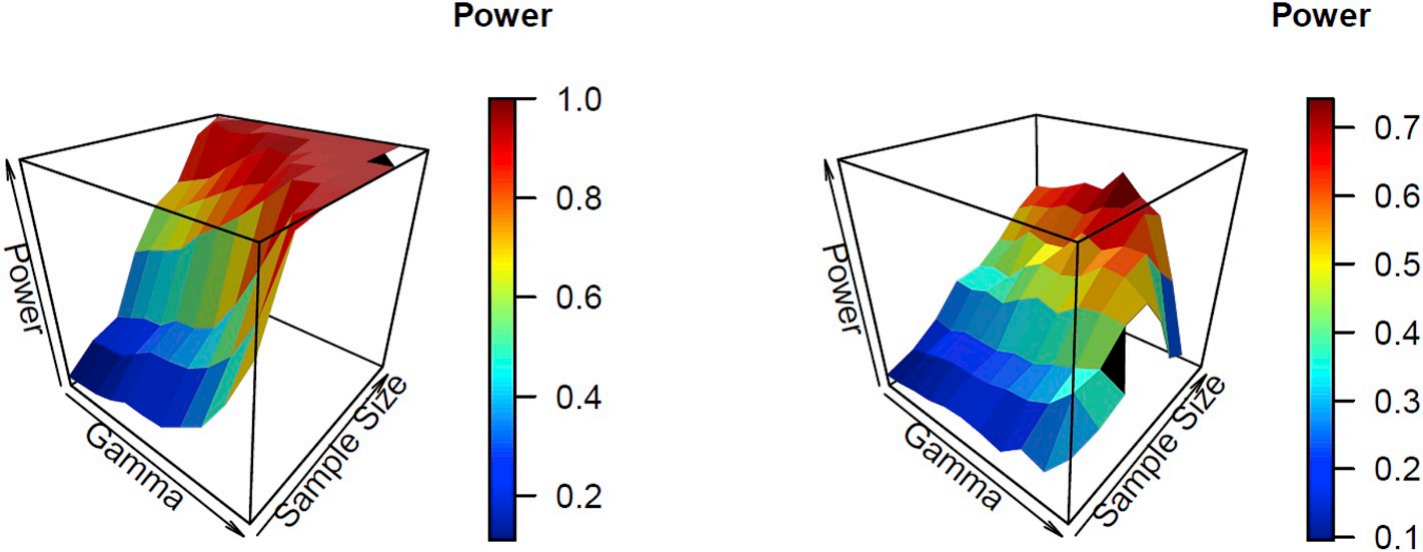
Surface plots of the estimated power for hypothesis test $\beta_{2}=0$ of $\gamma_{1}$ and sample size n. The left panel is for the Poisson time series with $\beta_{2}=-1$; the right panel is for the negative binomial time series with $\beta_{2}=-1$.

Table 3 and Table 4 show the estimated power for testing hypothesis (ii) $H_{0}:\beta_{2}=0$ for the Poisson and negative time series for model (2) and the pre-specified parameter values in the level change based on a significance level of 0.05. We considered $\beta_{2}=\pm 0.25,\;\pm 0.5,\;\pm 1$ for the Poisson time series in Table 3, and $\beta_{2}=\pm 1,\;\pm 2,\;\pm 3$ for the negative binomial time series in Table 4. For the Poisson models, the estimated power increased as $\gamma_{1}$, the sample size increased, or the values of the parameter became more significant. For negative binomial models, the results were similar to those of the Poisson models, but the estimated power was decreased for very large values of $\gamma_{1}$. The trends for the estimated power of $\gamma_{1}$ and sample size n are illustrated by the surface plots in Fig. 2.

**Table 3:** Estimated power testing $H_{0}:\beta_{2}=0$ for the Poisson time series with a conditional mean model LL (0,1) when $\beta_{2}$ = ±0.25, ±0.5, ±1 based on 200 simulated data sets and a statistical significance level of 0.05. The symbol “-” indicates more than one fourth of the data sets cannot be successfully generated.

$\gamma_{1}$	Sample size							
	18	24	32	48	56	64	80	96
$\beta_{2}$ = −1								
−0.9	0.05	0.16	0.21	0.49	0.67	0.74	0.93	0.99
−0.7	0.06	0.17	0.25	0.53	0.65	0.77	1.00	0.99
−0.5	0.07	0.18	0.29	0.56	0.72	0.80	0.97	1
−0.3	0.10	0.24	0.30	0.60	0.77	0.87	0.98	1
−0.1	0.15	0.30	0.37	0.69	0.82	0.93	1	1
0	0.15	0.31	0.38	0.75	0.87	0.98	1	1
0.1	0.16	0.33	0.43	0.82	0.90	0.97	1	1
0.3	0.23	0.39	0.57	0.90	0.99	1	1	1
0.5	0.30	0.49	0.75	0.99	1	1	1	1
0.7	0.46	0.67	0.95	1	1	1	1	1
0.9	0.68	0.98	1	1	1	1	–	–
$\beta_{2}$ = −0.5								
−0.9	0.04	0.09	0.07	0.21	0.25	0.33	0.45	0.63
−0.7	0.05	0.10	0.09	0.20	0.25	0.35	0.45	0.69
−0.5	0.05	0.12	0.12	0.25	0.26	0.44	0.59	0.77
−0.3	0.07	0.13	0.15	0.29	0.31	0.45	0.65	0.86
−0.1	0.11	0.18	0.12	0.35	0.42	0.52	0.77	0.89
0	0.11	0.17	0.16	0.39	0.46	0.55	0.80	0.99
0.1	0.12	0.19	0.20	0.43	0.46	0.66	0.88	1
0.3	0.15	0.23	0.25	0.60	0.73	0.87	0.98	1
0.5	0.20	0.31	0.39	0.84	0.94	1	1	1
0.7	0.22	0.41	0.75	1	1	1	1	1
0.9	0.56	0.95	1	1	1	–	–	–
$\beta_{2}$ = −0.25								
−0.9	0.03	0.05	0.06	0.12	0.13	0.11	0.16	0.22
−0.7	0.03	0.07	0.06	0.13	0.11	0.16	0.17	0.20
−0.5	0.04	0.08	0.07	0.14	0.14	0.14	0.19	0.25
−0.3	0.05	0.08	0.06	0.14	0.14	0.17	0.25	0.40
−0.1	0.07	0.11	0.07	0.16	0.20	0.17	0.29	0.47
0	0.07	0.11	0.09	0.18	0.20	0.28	0.34	0.54
0.1	0.09	0.13	0.10	0.14	0.22	0.27	0.43	0.63
0.3	0.12	0.17	0.12	0.22	0.33	0.45	0.60	0.93
0.5	0.13	0.15	0.25	0.43	0.56	0.74	0.98	1
0.7	0.18	0.27	0.38	0.86	0.98	1	1	1
0.9	0.33	0.72	0.99	1	–	–	–	–
$\beta_{2}$ = 0.25								
−0.9	0.04	0.06	0.06	0.09	0.10	0.13	0.13	0.28
−0.7	0.04	0.09	0.05	0.11	0.11	0.17	0.17	0.23
−0.5	0.04	0.08	0.07	0.11	0.14	0.15	0.26	0.28
−0.3	0.05	0.10	0.08	0.12	0.19	0.16	0.31	0.34
−0.1	0.05	0.08	0.07	0.10	0.13	0.23	0.32	0.48
0	0.07	0.10	0.10	0.15	0.17	0.19	0.35	0.50
0.1	0.06	0.10	0.10	0.13	0.17	0.28	0.49	0.72
0.3	0.09	0.11	0.10	0.25	0.40	0.45	0.76	0.97
0.5	0.12	0.16	0.24	0.42	0.71	0.81	0.99	1
0.7	0.13	0.27	0.49	0.94	0.99	1	1	1
0.9	0.10	0.64	1	–	–	–	–	–
$\beta_{2}$ = 0.5								
−0.9	0.06	0.12	0.10	0.22	0.28	0.33	0.54	0.66
−0.7	0.07	0.12	0.13	0.25	0.39	0.46	0.58	0.68
−0.5	0.07	0.13	0.14	0.30	0.35	0.48	0.63	0.84
−0.3	0.12	0.12	0.15	0.35	0.36	0.52	0.79	0.94
−0.1	0.11	0.17	0.22	0.44	0.50	0.64	0.91	1
0	0.11	0.165	0.21	0.46	0.53	0.73	0.92	1
0.1	0.13	0.20	0.20	0.50	0.67	0.78	0.97	1
0.3	0.16	0.21	0.43	0.79	0.86	0.96	0.99	1
0.5	0.23	0.39	0.65	0.97	1	1	1	1
0.7	0.43	0.72	0.95	1	1	1	1	1
0.9	0.80	1	0.99	–	–	–	–	–
$\beta_{2}$ = 1								
−0.9	0.22	0.32	0.47	0.81	0.89	0.94	0.99	1
−0.7	0.24	0.35	0.51	0.84	0.89	0.95	1	1
−0.5	0.27	0.42	0.54	0.84	0.94	0.99	1	1
−0.3	0.33	0.46	0.70	0.90	0.97	1	1	1
−0.1	0.36	0.51	0.73	0.97	0.99	1	1	1
0	0.42	0.59	0.80	0.98	0.99	1	1	1
0.1	0.48	0.66	0.82	1	1	1	1	1
0.3	0.64	0.83	0.96	1	1	1	1	1
0.5	0.79	0.97	1	1	1	1	1	1
0.7	0.99	1	0.97	1	1	1	1	1
0.9	0.99	0.97	0.98	–	–	–	–	–

**Table 4:** Estimated power testing $H_{0}:\beta_{2}=0$ for the negative binomial time series with a conditional mean model LL (0,1) when $\beta_{2}$ = ±1, ±2, ±3 based on 200 simulated data sets and a statistical significance level of 0.05. The symbol “-” indicates that more than one fourth of the data sets cannot be successfully generated.

$\gamma_{1}$	Sample size							
	18	24	32	48	56	64	80	96
$\beta_{2}$ = −3								
−0.9	0.02	0.05	0.32	0.91	0.96	1	1	1
−0.7	0.02	0.05	0.32	0.91	0.97	1	1	1
−0.5	0.03	0.05	0.34	0.94	0.97	1	1	1
−0.3	0.03	0.07	0.36	0.93	1	1	1	1
−0.1	0.03	0.08	0.40	0.94	0.99	1	1	1
0	0.03	0.08	0.42	0.93	0.99	1	1	1
0.1	0.03	0.11	0.43	0.93	0.99	1	1	1
0.3	0.02	0.21	0.51	0.93	1	1	1	1
0.5	0.07	0.22	0.53	0.97	1	0.99	1	1
0.7	0.11	0.40	0.66	0.95	0.96	0.96	0.94	0.87
0.9	0.22	0.50	0.66	–	–	–	–	–
$\beta_{2}$ = −2								
−0.9	0.04	0.15	0.34	0.76	0.86	0.89	0.98	1
−0.7	0.05	0.18	0.39	0.78	0.87	0.94	0.99	1
−0.5	0.06	0.20	0.38	0.83	0.85	0.94	0.99	1
−0.3	0.07	0.21	0.42	0.84	0.96	0.95	0.98	1
−0.1	0.07	0.22	0.47	0.86	0.95	0.98	1	1
0	0.07	0.24	0.50	0.84	0.95	0.97	1	1
0.1	0.09	0.28	0.52	0.88	0.94	0.98	0.99	1
0.3	0.12	0.34	0.58	0.87	0.93	0.98	1	1
0.5	0.13	0.43	0.57	0.90	0.95	0.98	0.98	0.96
0.7	0.25	0.50	0.67	0.93	0.92	0.92	0.90	0.54
0.9	0.35	0.52	0.61	–	–	–	–	–
$\beta_{2}$ = −1								
−0.9	0.06	0.11	0.20	0.34	0.36	0.45	0.57	0.65
−0.7	0.07	0.14	0.18	0.33	0.39	0.47	0.61	0.66
−0.5	0.09	0.17	0.22	0.36	0.37	0.55	0.62	0.69
−0.3	0.10	0.19	0.22	0.34	0.46	0.52	0.66	0.73
−0.1	0.13	0.19	0.22	0.40	0.51	0.53	0.64	0.72
0	0.14	0.20	0.26	0.45	0.49	0.63	0.70	0.82
0.1	0.13	0.23	0.26	0.42	0.52	0.58	0.72	0.73
0.3	0.11	0.24	0.31	0.49	0.60	0.63	0.72	0.67
0.5	0.20	0.29	0.35	0.51	0.59	0.63	0.49	0.38
0.7	0.17	0.40	0.40	0.50	0.52	0.36	0.09	0.02
0.9	0.31	0.31	0.35	–	–	–	–	–
$\beta_{2}$ = 1								
−0.9	0.15	0.23	0.28	0.42	0.44	0.52	0.56	0.71
−0.7	0.20	0.21	0.29	0.40	0.47	0.47	0.66	0.73
−0.5	0.18	0.24	0.29	0.41	0.46	0.52	0.66	0.77
−0.3	0.21	0.26	0.39	0.49	0.54	0.62	0.69	0.78
−0.1	0.17	0.32	0.39	0.56	0.59	0.62	0.72	0.82
0	0.27	0.35	0.38	0.54	0.63	0.69	0.70	0.81
0.1	0.24	0.27	0.39	0.56	0.61	0.59	0.73	0.80
0.3	0.29	0.39	0.40	0.63	0.62	0.68	0.72	0.71
0.5	0.32	0.42	0.56	0.62	0.62	0.67	0.60	0.58
0.7	0.42	0.49	0.61	0.58	0.52	0.50	–	–
0.9	0.23	0.27	–	–	–	–	–	–
$\beta_{2}$ = 2								
−0.9	0.53	0.64	0.75	0.88	0.94	0.93	0.98	0.99
−0.7	0.50	0.67	0.80	0.94	0.95	0.98	0.99	1
−0.5	0.58	0.67	0.81	0.94	0.96	0.99	1	1
−0.3	0.58	0.72	0.84	0.98	0.99	0.98	1	1
−0.1	0.67	0.77	0.86	0.98	1.00	0.99	1	1
0	0.67	0.78	0.89	0.98	0.99	0.99	1.	1
0.1	0.67	0.79	0.93	0.99	0.97	0.98	0.99	0.97
0.3	0.79	0.82	0.88	0.92	0.97	0.97	0.94	0.93
0.5	0.78	0.88	0.84	0.85	0.85	0.84	0.77	0.73
0.7	0.70	0.78	0.74	0.74	–	–	–	–
0.9	–	–	–	–	–	–	–	–
$\beta_{2}$ = 3								
−0.9	0.77	0.92	0.95	0.99	0.99	1	1	1
−0.7	0.85	0.87	0.98	0.99	1	1	0.99	1
−0.5	0.82	0.95	0.99	1	1	1	1	1
−0.3	0.89	0.98	1.00	1	1	1	1	1
−0.1	0.89	0.97	1.00	1	1	1	1	1
0	0.93	0.96	0.99	1	1.00	1	1	1
0.1	0.92	0.98	0.99	1.00	1.00	1	1	1
0.3	0.93	0.97	0.95	0.96	0.97	0.97	0.96	0.97
0.5	0.89	0.94	0.93	0.88	0.91	0.86	0.83	–
0.7	0.77	0.79	0.71	–	–	–	–	–
0.9	–	–	–	–	–	–	–	–

**Fig. 3:**
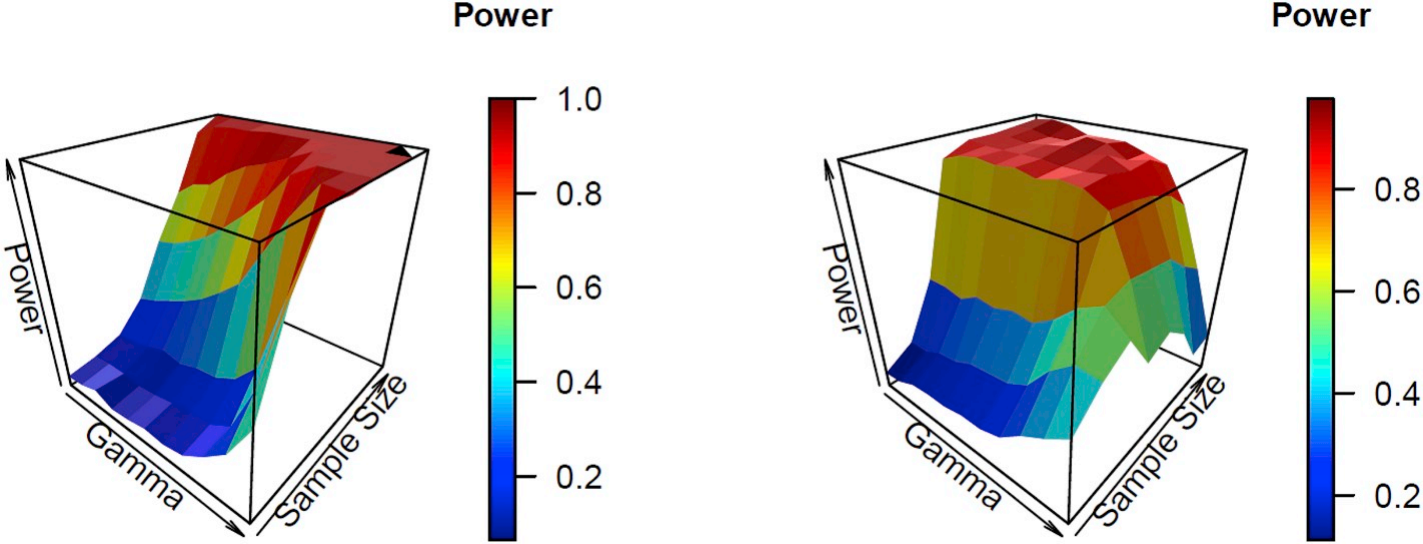
Surface plots of the estimated power for hypothesis test $\beta_{3}=0$ of $\gamma_{1}$ and sample size n. The left panel is for the Poisson time series with $\beta_{3}=-0.1$; the right panel is for the negative binomial time series with $\beta_{3}=-0.2$.

Table 5 and Table 6 show the estimated power testing $H_{0}:\beta_{3}=0$ for the Poisson and negative time series with model (2) and the pre-specified values of the trend change parameter based on a significance level of 0.05. We considered $\beta_{3}=\;\pm 0.01,\;\pm 0.05,\;\pm 0.10$ for the Poisson time series in Table 5, and $\beta_{3}=\pm 0.05,\;\pm 0.1,\;\pm 0.25$ for the negative binomial time series in Table 6. Similar to the previous test, the estimated power increased as $\gamma_{1}$, the sample size increased, or the values of the parameter became more significant for the Poisson time series. For the negative binomial time series, again, the estimated power increased first and then decreased as $\gamma_{1}$ increased. This phenomenon can be more clearly observed for large values of the parameter. Further, when the value of the parameter was negative, the estimated power increased first and then decreased as the values of the parameter decreased. The difference in the estimated power between the parameter values of the opposite signs is due to the fact that count data are defined based on the non-negative support. Thus, models are built on the logarithm of the conditional mean of the responses. The trends of the estimated power of $\gamma_{1}$ and sample size n are illustrated by the surface plots in Fig. 3.

**Table 5:** Estimated power testing $H_{0}:\beta_{3}=0$ for the Poisson time series with a conditional mean model LL (0,1) when $\beta_{3}$ = ±0.01, ±0.05, ±0.10 based on 200 simulated data sets and a statistical significance level of 0.05. The symbol “-” indicates that more than one fourth of the data sets cannot be successfully generated.

$\gamma_{1}$	Sample size							
	18	24	32	48	56	64	80	96
$\beta_{3}$ = −0.10								
−0.9	0.07	0.10	0.23	0.68	0.93	0.98	1	1
−0.7	0.08	0.11	0.26	0.76	0.97	1	1	1
−0.5	0.09	0.13	0.27	0.80	0.97	1	1	1
−0.3	0.09	0.12	0.27	0.82	0.99	1	1	1
−0.1	0.09	0.17	0.32	0.90	0.99	1	1	1
0	0.12	0.17	0.34	0.94	1	1	1	1
0.1	0.13	0.17	0.38	0.96	1	1	1	1
0.3	0.15	0.24	0.51	0.98	1	1	1	1
0.5	0.26	0.37	0.69	1	1	1	1	1
0.7	0.38	0.53	0.93	1	1	1	1	1
0.9	0.72	0.93	1	1	1	1	1	–
$\beta_{3}$ = −0.05								
−0.9	0.05	0.07	0.11	0.24	0.49	0.69	0.94	1
−0.7	0.06	0.08	0.11	0.27	0.51	0.75	0.98	1
−0.5	0.09	0.08	0.14	0.31	0.55	0.77	0.99	1
−0.3	0.05	0.07	0.17	0.35	0.60	0.84	0.99	1
−0.1	0.06	0.08	0.15	0.40	0.67	0.89	1	1
0	0.08	0.10	0.18	0.47	0.75	0.92	1	1
0.1	0.07	0.11	0.19	0.55	0.84	0.96	1	1
0.3	0.09	0.12	0.24	0.71	0.95	1	1	1
0.5	0.16	0.18	0.35	0.87	1	1	1	1
0.7	0.25	0.41	0.68	1	1	1	1	1
0.9	0.43	0.79	1	0.99	1	1	1	–
$\beta_{3}$ = −0.01								
−0.9	0.05	0.07	0.07	0.06	0.05	0.05	0.13	0.19
−0.7	0.05	0.09	0.05	0.09	0.07	0.09	0.15	0.19
−0.5	0.06	0.07	0.06	0.06	0.08	0.09	0.20	0.21
−0.3	0.05	0.09	0.08	0.07	0.07	0.11	0.16	0.33
−0.1	0.07	0.09	0.06	0.07	0.08	0.11	0.14	0.40
0	0.08	0.09	0.08	0.08	0.09	0.12	0.19	0.38
0.1	0.08	0.10	0.10	0.09	0.10	0.16	0.26	0.48
0.3	0.09	0.09	0.08	0.12	0.11	0.19	0.34	0.72
0.5	0.12	0.09	0.09	0.17	0.21	0.28	0.66	0.95
0.7	0.14	0.15	0.14	0.24	0.42	0.70	1	0.99
0.9	0.12	0.14	0.13	0.95	–	–	–	–
$\beta_{3}$ = 0.01								
−0.9	0.07	0.07	0.06	0.08	0.10	0.08	0.14	0.19
−0.7	0.06	0.09	0.09	0.07	0.07	0.15	0.13	0.28
−0.5	0.06	0.10	0.07	0.10	0.06	0.11	0.19	0.25
−0.3	0.05	0.11	0.06	0.09	0.09	0.12	0.12	0.31
−0.1	0.09	0.10	0.09	0.09	0.07	0.14	0.18	0.37
0	0.10	0.11	0.09	0.11	0.07	0.12	0.24	0.47
0.1	0.08	0.12	0.10	0.09	0.12	0.15	0.34	0.50
0.3	0.08	0.13	0.09	0.12	0.15	0.21	0.41	0.64
0.5	0.11	0.10	0.12	0.16	0.25	0.36	0.64	0.90
0.7	0.13	0.12	0.17	0.35	0.47	0.61	0.80	0.99
0.9	0.16	0.11	0.06	–	–	–	–	–
$\beta_{3}$ = 0.05								
−0.9	0.08	0.10	0.16	0.45	0.50	0.78	0.98	1
−0.7	0.09	0.11	0.20	0.39	0.55	0.83	0.99	1
−0.5	0.09	0.12	0.20	0.49	0.66	0.82	1	1
−0.3	0.10	0.14	0.23	0.46	0.73	0.88	0.99	1
−0.1	0.12	0.15	0.17	0.58	0.78	0.94	1	1
0	0.13	0.18	0.24	0.61	0.79	0.97	1	1
0.1	0.13	0.18	0.25	0.71	0.88	0.98	1	1
0.3	0.12	0.16	0.33	0.82	0.94	0.98	1	1
0.5	0.13	0.22	0.49	0.87	0.97	0.99	0.99	0.97
0.7	0.22	0.43	0.67	0.98	0.99	0.99	0.98	–
0.9	0.22	0.30	0.83	–	–	–	–	–
$\beta_{3}$ = 0.10								
−0.9	0.13	0.19	0.36	0.90	0.99	1	1	1
−0.7	0.17	0.18	0.47	0.92	0.99	1	1	1
−0.5	0.16	0.23	0.43	0.95	1	1	1	1
−0.3	0.17	0.27	0.50	0.95	1	1	1	1
−0.1	0.19	0.25	0.53	0.97	1	1	1	1
0	0.17	0.31	0.56	0.97	1	1	1	1
0.1	0.20	0.32	0.64	0.99	1	1	1	1
0.3	0.19	0.40	0.75	0.98	1	1	1	1
0.5	0.29	0.58	0.84	1	1	1	0.99	1
0.7	0.48	0.71	0.88	0.98	0.98	–	–	–
0.9	0.38	0.90	0.89	–	–	–	–	–

**Table 6:** Estimated power testing $H_{0}:\beta_{3}=0$ for the negative binomial time series with a conditional mean model LL (0,1), when $\beta_{3}$ = ±0.05, ±0.10, ±0.25 based on 200 simulated data sets and a statistical significance level of 0.05. The symbol “-” indicates that more than one fourth of the data sets cannot be successfully generated.

$\gamma_{1}$	Sample size							
	18	24	32	48	56	64	80	96
$\beta_{3}$ = −0.25								
−0.9	0.07	0.15	0.41	0.91	0.96	0.96	0.97	0.98
−0.7	0.08	0.15	0.41	0.94	0.95	0.96	0.97	0.99
−0.5	0.12	0.19	0.39	0.95	0.95	0.95	0.95	0.98
−0.3	0.13	0.20	0.44	0.94	0.95	0.96	0.95	0.95
−0.1	0.13	0.24	0.43	0.95	0.95	0.97	0.98	0.95
0	0.15	0.25	0.46	0.93	0.94	0.95	0.97	0.95
0.1	0.15	0.26	0.45	0.95	0.94	0.97	0.92	0.92
0.3	0.18	0.31	0.51	0.94	0.95	0.93	0.94	0.89
0.5	0.26	0.43	0.60	0.89	0.88	0.86	0.82	0.74
0.7	0.32	0.51	0.65	0.68	0.66	0.64	0.55	0.48
0.9	0.39	0.50	0.57	0.39	0.44	0.35	0.16	0.18
$\beta_{3}$ = −0.10								
−0.9	0.08	0.09	0.19	0.51	0.73	0.86	1	1
−0.7	0.08	0.10	0.20	0.59	0.78	0.89	0.99	1
−0.5	0.09	0.12	0.19	0.55	0.80	0.92	1	1
−0.3	0.10	0.11	0.23	0.60	0.82	0.95	1	1
−0.1	0.08	0.14	0.24	0.61	0.83	0.93	1	1
0	0.09	0.15	0.23	0.70	0.85	0.95	1	1
0.1	0.11	0.18	0.27	0.72	0.84	0.99	1	1
0.3	0.10	0.15	0.28	0.71	0.89	0.99	1	1
0.5	0.17	0.26	0.37	0.73	0.87	0.95	1	0.99
0.7	0.21	0.28	0.37	0.70	0.74	0.74	0.73	0.66
0.9	0.30	0.36	0.34	0.39	–	–	–	–
$\beta_{3}$ = −0.05								
−0.9	0.09	0.09	0.10	0.18	0.27	0.36	0.71	0.89
−0.7	0.06	0.09	0.10	0.16	0.28	0.45	0.72	0.93
−0.5	0.09	0.08	0.10	0.21	0.28	0.44	0.72	0.93
−0.3	0.09	0.10	0.12	0.26	0.37	0.49	0.82	0.93
−0.1	0.09	0.12	0.12	0.27	0.40	0.54	0.79	1.00
0	0.10	0.11	0.14	0.23	0.44	0.58	0.84	0.98
0.1	0.11	0.10	0.17	0.26	0.39	0.56	0.87	0.99
0.3	0.12	0.12	0.12	0.29	0.39	0.57	0.84	0.96
0.5	0.17	0.16	0.18	0.32	0.43	0.60	0.81	0.93
0.7	0.15	0.14	0.18	0.37	0.38	0.45	0.53	0.48
0.9	0.21	0.17	0.17	0.14	0.07	–	–	–
$\beta_{3}$ = 0.05								
−0.9	0.10	0.11	0.09	0.16	0.30	0.30	0.49	0.66
−0.7	0.11	0.09	0.10	0.18	0.25	0.33	0.56	0.80
−0.5	0.12	0.13	0.11	0.23	0.31	0.40	0.65	0.81
−0.3	0.14	0.13	0.12	0.19	0.35	0.44	0.63	0.80
−0.1	0.10	0.10	0.12	0.24	0.29	0.36	0.61	0.74
0	0.15	0.09	0.14	0.24	0.26	0.39	0.53	0.72
0.1	0.10	0.15	0.13	0.20	0.27	0.33	0.49	0.66
0.3	0.13	0.11	0.16	0.16	0.19	0.27	0.31	0.31
0.5	0.17	0.13	0.10	0.16	0.14	0.12	0.15	0.12
0.7	0.14	0.08	0.08	0.04	0.04	0.04	–	–
0.9	0.08	0.04	0.02	–	–	–	–	–
$\beta_{3}$ = 0.10								
−0.9	0.10	0.11	0.23	0.49	0.67	0.77	0.93	0.99
−0.7	0.11	0.13	0.25	0.53	0.69	0.82	0.93	0.98
−0.5	0.09	0.22	0.18	0.51	0.75	0.85	0.95	0.97
−0.3	0.14	0.18	0.26	0.56	0.72	0.84	0.93	0.98
−0.1	0.11	0.18	0.26	0.54	0.66	0.76	0.81	0.84
0	0.14	0.19	0.23	0.53	0.64	0.74	0.76	0.74
0.1	0.16	0.15	0.21	0.47	0.50	0.59	0.65	0.57
0.3	0.12	0.17	0.23	0.28	0.30	0.32	0.36	0.36
0.5	0.15	0.14	0.13	0.17	0.18	0.13	–	–
0.7	0.12	0.08	0.04	–	–	–	–	–
0.9	0.07	–	–	–	–	–	–	–
$\beta_{3}$ = 0.25								
−0.9	0.31	0.45	0.68	0.94	0.94	0.95	0.96	0.94
−0.7	0.24	0.42	0.64	0.97	0.93	0.93	0.91	0.91
−0.5	0.19	0.41	0.72	0.89	0.90	0.91	0.87	0.82
−0.3	0.26	0.40	0.67	0.79	0.79	0.80	0.72	0.69
−0.1	0.27	0.36	0.56	0.62	0.61	0.62	0.56	–
0	0.23	0.33	0.43	0.56	0.53	0.48	0.49	–
0.1	0.22	0.29	0.45	0.38	0.41	0.34	–	–
0.3	0.20	0.27	0.22	0.19	0.20	–	–	–
0.5	0.15	0.13	0.10	–	–	–	–	–
0.7	0.07	–	–	–	–	–	–	–
0.9	–	–	–	–	–	–	–	–

For large absolute values of $\gamma_{1}$, the time series were more likely to explode, i.e., the data in the certain time series can increase (or decrease) so fast that the computer program cannot generate values over a certain threshold because of this rapid expansion. It was also often impossible to generate a time series with the desired sample size. This situation usually happened for large sample sizes. Estimations do not exist for these exploded models, since data cannot be successfully generated, so the estimated powers are marked with the symbol “-” in the tables when more than one fourth of the simulations (more than 50 times) could not generate a time series with the specified length.

## 5 Discussion

ITS is a powerful yet simple quasi-experimental design that has been widely applied to many population-based public health and health service intervention studies ([2,7]). In this article, we studied the models of ITS design for count outcomes. More specifically, we discussed low order log-linear models for ITS design, a special type of observation-driven model, with two distribution specifications (Poisson and negative binomial). Our study was motivated by the STRIDE study, which was designed based on the state-of-the-art power calculation method of the two-arm two-phase ITS design of continuous outcomes (the rate of African American and Latino participants recruited) proposed by Zhang et al. [6]. Because we were also interested in the number of African American and Latino participants recruited and the total number of participants, similar power calculation method using ITS design for count outcomes needed to be investigated. Herein, a simulation-based method was applied to demonstrate the power of hypothesis tests on level change, trend change, and the change of both (the sum of the level change and trend change) under different values of parameters, sample sizes, and autocorrelation coefficients ($\gamma_{1}$) under pre-specified conditions. We focused our attention on single-arm ITS studies. Tests for two-arm ITS studies require future investigation. As anticipated, for Poisson models, the estimated power increased as $\gamma_{1}$, the sample size increased, or the values of the parameter became more significant. For the negative binomial method, the estimated power increased as the sample size increased, or values of the parameter became more significant. However, the change of power showed a U-shape pattern as $\gamma_{1}$ increased for tests on level change and trend change and also increased as $\gamma_{1}$ increased for the tests of the total change. Further, summarizing the results across the six tables, the power of the hypothesis tests with the same level of parameter values can vary widely depending on the type of tests (level, trend, or both) and the model specifications.

Like most ITS designs, our simulation-based power and sample size calculations were based upon models at the aggregated data level. For instance, in the STRIDE study, the aggregate number of participants of African Americans and Latino descent will be collected weekly. However, this type of analysis will not only lose information when individual level data are unavailable, but can also give an incomplete conclusion if the total number of participants increases simultaneously. Thus, although aggregate level ITS designs are the common practice, power and effect size calculations based on such an approach only consider the number of time tables, but not the number of observations at each time window. For this reason, individual level ITS designs for count data or ITS designs that account for the number of observations at each time window need to be further investigated.

This study has several limitations. Firstly, we only considered observation-driven ITS models. Previous studies suggested that parameter-driven models are usually more complicated and computationally intensive because full likelihood of these models involve high-dimensional integration. Yet parameter-driven models have better interpretability for their parameters than observation-driven models. Thus, the performance of parameter-driven models for ITS design, based on count outcomes, needs to be further studied and compared with our proposed models. Secondly, it may be too simplistic to assume that an intervention is implemented at a single time point. Using the STRIDE study as an example, it is reasonable to assume that a “ramp-up” period is required to allow the research assistants to complete their training and for the intervention to achieve full implementation. Further, the study contains a comparison group. Although the ITS study may still be valid with the absence of a control study ([7]), and adapt the three-phase design to a two-phase design ([35]), the strength of the inference will be weaker. Therefore, the power and effect size calculations of count outcomes for more complicated models like two-arm three-phase ITS design should be further investigated. Thirdly, as mentioned above, the integrated level ITS design does not consider the number of individuals at each timetable. Using the STRIDE study as an example, this limitation may yield incomplete conclusions, since we expect an increase in the number of African Americans, Latinos, and total participants. Individual-level ITS design could be a reasonable approach to overcome this issue, though only a few health policy studies ([36]) have taken such an approach. Fourthly, excessive zeros are an issue in health policy studies, including the STRIDE study. Our ongoing research seeks to extend our work to zero-inflated Poisson or zero-inflated negative binomial models.

## 6 Conclusions

Sample size and power calculations were conducted for ITS studies of count outcomes using an observation-driven model through the simulation-based methods presented in this article. Results varied among the different model specifications and the target of the study (i.e., investigating level change, trend change, or both).

## Author's contributions

BZ, WL, and SY presented the research idea. WL performed the numerical simulation. BZ and SY wrote the original manuscript with support from MAP, EJR, MID, and CL. The STRIDE principal investigators, KGS, PAH, SCL, and JJA, motivated the research idea and helped supervise the project.

## Declaration of competing interest

None.

## Appendix

This appendix includes the R program for conducting sample size and power calculations for the interrupted time series analysis of the count outcomes presented in this article.

*library(tscount)*
*ITSC.single.group = function(nsmp=18, bet.inter, bet.time, bet.x1, bet.timex1,*
  *gam, time, time.intrv1, y0=0, mu0=3){*
*# nsmp: the sample size (the number of time points)*
*# pchi.14: the upper quantile of chi-square (df)*
*# time: a vector of time (start.time:final.time)*
*# time.intrv1: indicator for onset of the intervention in time.*
*# Regression coefficients:*
*# bet.inter: intercept coefficient*
*# bet.x1: the coefficient for the binary indicator for the second phase of the study*
*# bet.time: the coefficient for time*
*# bet.timex1: the coefficient for the interaction of x1 and time*
*# gam: the coefficient* γ
*# mu0: the coefficient* μ
*pchi.14 = qchisq(0.95, 2)*
*pchi.24 = qchisq(0.95, 1)*
*bet = c(bet.inter, bet.time, bet.x1, bet.timex1 )*
*x1 = c(rep(0,time.intrv1), rep(1,nsmp-time.intrv1))*
*logtime = time*
*logtime1 = time-time.intrv1*
*#--------- generate data ---------#*
*x.t= model.matrix( ∼ logtime + x1 + logtime1:x1-1 )*
*eta = apply(cbind(1,x.t), 1, function(s){sum(s*bet)})*
*mu.lag = mu0*
*y = rep(NA, nsmp+1)*
*y[1] = y0*
*for (i in 2:(nsmp+1)){*
  *e.lag = gam*log(y[i-1]+1)*
  *mu.lag = exp(eta[i-1] + e.lag)*
  *y[i] = rpois(1,mu.lag) # or rnbinom*
*}*
*#--------- fit and test ---------#*
*f.fit.LLA = function(y, desg, y0, mu0,tst){*
  *fit = try(tsglm(y, model=list(past_obs=1), xreg=desg,*
  *link = "log", distr = "poisson" ),silent = T) # or "distr=nbinom"*
  *if(class(fit)=="try-error") tst=-1*
  *if (tst ==4){*
    *est.q = coef(fit)[4:5] #est*
    *covmatr = try(solve(fit$info.matrix_corrected)[4:5,4:5],silent = T)*
    *se.q = try(se(fit)$se[4:5],silent = T) #SE =sqrt(diag(covmatr))*
*if(class(se.q)=="try-error"|class(covmatr)=="try-error"){*
*covmatr=NA*
*se.q = NA*
*}*
*Nloglik.tr = scoring(fit)[1] # log-likelihood*
*}else{*
*if(tst==-1){*
*est.q=NA*
*se.q=NA*
*covmatr=NA*
*Nloglik.tr=NA*
*}else{*
*est.q=NA*
*se.q=NA*
*covmatr=NA*
*Nloglik.tr = scoring(fit)[1] # log-likelihood*
*}*
*}*
*list(est.q = est.q, se.q=se.q, Nloglik.tr=Nloglik.tr, covmatr = covmatr)*
*}*
*fit4 = f.fit.LLA(y=y[-1], desg=x.t, y0=y[1], mu0=mu0, tst=4)*
*est = fit4$est.q*
*se.c = fit4$se.q*
*dif = est/se.c*
*if(any(is.na(dif))){*
  *rej = rep(NA,3)*
*}else{*
  *t23 = dif^2*
  *est1 = matrix(est,nrow=1)*
  *t1 = est1%*%solve(fit4$covmatr)%*%t(est1)*
  *rej = (c(t1> pchi.14 ,t23> pchi.24))*
*}*
*rej*
*}*
